# Nephrotoxicity as a Complication of Chemotherapy and Immunotherapy in the Treatment of Colorectal Cancer, Melanoma and Non-Small Cell Lung Cancer

**DOI:** 10.3390/ijms22094618

**Published:** 2021-04-28

**Authors:** Joanna Jagieła, Piotr Bartnicki, Jacek Rysz

**Affiliations:** Department of Nephrology, Hypertension and Family Medicine, Medical University of Łódź, Żeromskiego 113, 90-549 Łódź, Poland; joanna.jagiela.1@umed.lodz.pl (J.J.); piotr.bartnicki@umed.lodz.pl (P.B.)

**Keywords:** acute kidney injury, immunotherapy, chemotherapy

## Abstract

Acute kidney injury is a common complication of many medical procedures, including those used in cancer treatment. Both chemotherapy and immunotherapy may result in deterioration of kidney function, which may lead to an increase in mortality among patients with cancer. Antineoplastic agents can affect any element of the nephron, leading to the appearance of clinical symptoms such as proteinuria, hypertension, electrolyte disorders, glomerulonephritis, acute and chronic interstitial nephritis and acute kidney injury. The medical literature describing renal complications occurring during chemotherapeutic and immunotherapeutic treatment in neoplasms, such as colorectal cancer, non-small cell lung cancer and melanoma, was analysed. The immune system plays an important role in controlling the development of neoplasms and fighting them. Oncological treatment algorithms include immunotherapy as monotherapy, combined with chemotherapy or chemotherapy as monotherapy. In the treatment of the above-mentioned neoplasms immunotherapeutics are used, such as checkpoint inhibitors (CPI) (i.e., ipilimumab, pembrolizumab, nivolumab, atezolizumab), vascular endothelial growth factor (VEGF) inhibitors (i.e., bevacizumab, ramucirumab) and a variety of chemotherapeutic agents (irinotecan, capecitabine, oxaliplatin, gefitinib, erlotinib, gemcitabine, cisplatin, paclitaxel, carboplatin, doclitaxel, vinorelbine, topotecan, etoposide). In our article, we focused on the number and type of renal complications as well as on the time of their manifestation when using specific treatment regimens. Our analysis also includes case reports. We discussed treatment of immunological complications and adjustments of the dose of chemotherapeutic agents depending on the creatinine clearance. Analysing the data from the literature, when two immunotherapeutic agents are used together, the number of recorded renal complications increases. Bevacizumab and ramucirumab are the cause of the largest number of renal complications among the immunotherapeutic agents described above. Cisplatin is the best-described substance with the greatest nephrotoxic potential among the chemotherapeutic agents. Crucial for renal complications are also cancer stage, previous chemotherapy and other risk factors of AKI such as age, comorbidities and medications used. Due to the described complications during oncological treatment, including kidney damage, it seems necessary to elaborate standards of cooperation between oncologists and nephrologists both during and after treatment of a patient with cancer. Therefore, it is necessary to conduct further research and develop algorithms for management of a cancer patient, especially during such an intensive progress in oncology.

## 1. Introduction

The kidneys are key organs for the proper functioning of the body and for the maintenance of homoeostasis. Their role is, among other things, to remove endogenous metabolic products as well as to eliminate medicines and toxins. Therefore, they are exposed to injury caused by the nephrotoxic effects of numerous substances [1], including drugs used in oncology. Kidney injury can also result from other medical treatments, such as radiation therapy. Kidney injury is a serious complication resulting both from the progression of the neoplastic disease itself and the method of treatment. Treatment-induced nephrotoxicity contributes to increased morbidity and mortality in cancer patients [2]. Over the years, the spectrum of kidney disease in cancer patients has changed, mainly as a result of modifications to the chemotherapy regimens [3] and the introduction of immunotherapy. Antineoplastic drugs can damage the renal tubules, glomeruli, parenchyma and blood vessels, leading to a wide range of complications, ranging from an asymptomatic increase in serum creatinine to acute kidney injury (AKI). In the following article, we used standardised criteria for adverse events in the context of renal complications. In relation to Common Terminology Criteria for Adverse Events (from v3.0 to v5.0) and Common Toxicity Criteria (from v1.0 to v2.0), we described acute kidney injury, elevated creatinine and proteinuria according to corresponding grades. For the increase in creatinine levels, we used the following grades: grade 1 (>1–1.5 × baseline; >ULN (upper limit of normal)-1.5 × ULN), grade 2 (>1.5–3.0 × baseline; >1.5–3.0 × ULN), grade 3 (>3.0 baseline; >3.0–6.0 × ULN) and grade 4 (>6.0 × ULN). For acute kidney injury, the grading scale is as follows: grade 1 (creatinine level increase of >0.3 mg/dL; creatinine 1.5–2.0 × above baseline), grade 2 (creatinine 2–3 × above baseline), grade 3 (creatinine >3 × baseline or >4.0 mg/dL; hospitalisation indicated), grade 4 (life-threatening consequences; dialysis indicated) and grade 5 (death). Regarding proteinuria, the scale is as follows: grade 1 (1+ proteinuria; urinary protein <1.0 g/24 h), grade 2 (2+ or 3+ proteinuria; urinary protein 1.0–3.4 g/24 h) and grade 3 (4+ proteinuria, urinary protein ≥3.5 g/24 h) [4]. In this review, renal complications that occur during chemotherapeutic and immunotherapeutic treatments of selected neoplasms, such as colorectal cancer, non-small cell lung carcinoma and melanoma, will be discussed.

## 2. Nephrotoxicity in the Immunological Treatment of Neoplasms

The immune system plays an important role in controlling the development of and fighting neoplasms. Therefore, immunotherapy is perceived as the main component of the treatment of cancer patients in the future [5]. Checkpoint inhibitors (CPI) deserve special attention. These are antibodies directed against cytotoxic T-lymphocyte-associated protein 4 (CTLA-4) (i.e., Ipilimumab), programmed cell death protein 1 (PD-1) (i.e., pembrolizumab, nivolumab) and programmed death-ligand 1 (PD-L1) (i.e., atezolizumab) [6].

CTLA-4 activity includes modulation of the primary immune response during activation of T cells by antigen-presenting cells (APCs). Upon activation of T cells, CTLA-4 migrates, and then competes with CD28 for binding to B7 ligands on APC. Binding of CTLA-4 to B7 ligands inhibits the further activation of T cells, limiting the duration of their activity. This mechanism regulates the intensity and duration of the primary immune response. In the event of a neoplastic process, inhibition of CTLA-4 may lead to further activation of more T cells, which may result in a more effective anti-tumour response [7]. The main role of PD-1 is to reduce the activity of T cells in peripheral tissues during the inflammatory response caused by infection and to limit autoimmunity. This translates into the main mechanism of immunity in the tumour microenvironment [8]. Since many tumour cells express PD-L1 (constitutively or after IFNγ induction), blocking PD-1 by antibodies improves T cell activation and reduces tumour progression [9].

Other substances used in the treatment of neoplasms are vascular endothelial growth factor (VEGF) inhibitors (i.e., Bevacizumab, Ramucirumab). VEGF is a glycoprotein responsible for the angiogenesis process. It binds two receptors, VEGF-1 and VEGF-2, which are expressed on vascular endothelial cells. This factor is involved in angiogenesis during embryonic development and wound healing. It is the main mediator of angiogenesis in neoplasms, where it is regulated by the expression of oncogenes, growth factors and hypoxia [10]. The use of an antibody targeted at a VEGF ligand or vascular endothelial growth factor (VEGFR) will result in its binding and sequestration [11].

Another mechanism used in cancer therapy is the action of antibodies directed against the extracellular domain of the epidermal growth factor receptor (EGFR) (i.e., cetuximab, panitumumab). The physiological function of EGFR is to regulate the development of epithelial tissue and maintain homoeostasis [12]. In the event of the overexpression of EGFR or increased activity of EGFR signalling pathways, the cell may transform into a state of constant unregulated proliferation, increasing the number of malignant cells [13]. Anti-EGFR antibodies block ligand binding and receptor degradation, thereby inhibiting the downstream signalling pathway [14].

### 2.1. Nephrotoxicity in the Immunotherapy of Melanoma

Malignant melanoma is a neoplasm that originates from pigment cells—melanocytes—which develop from the nervous tissue of the integuments. It is now believed that the development of melanoma is influenced by both genetic susceptibility and environmental exposure. While the incidence of numerous types of neoplasm is declining, the incidence of melanoma continues to rise [15]. Treatment options for melanoma include surgical resection, chemotherapy, photodynamic therapy, immunotherapy, biochemotherapy and targeted therapy. The choice of the type of treatment (monotherapy or combination therapy) depends on the patient’s health, as well as the stage and location of the tumour [16]. In the present review, the authors will focus on renal complications associated with immunosuppressive therapy.

Nivolumab is widely used in the treatment of melanoma both as a monotherapy and in combination with ipilimumab. In the trial conducted by Robert et al. (a randomised, controlled, phase III, double-blind trial), patients with confirmed, inoperable, previously untreated stage III or IV melanoma without BRAF mutation were enrolled: 418 patients were allocated to two groups: I (*n* = 210), nivolumab was used at a dose of 3 mg/kg every 2 weeks, and II (*n* = 208), dacarbazine was used at a dose of 1000 mg/m^2^ of body surface area every 3 weeks. The follow-up lasted up to 16.7 months. Out of 205 patients, 4 (1.9%) were reported as suffering from renal complications, and only 1 was classified as suffering from grade 3–4 complications. All these complications were considered to be potentially caused by an immunological process. The patient’s symptoms resolved within 6.1 weeks (after the treatment in line with the guidelines was applied) [17]. Topalian and co-authors published a study in which 107 patients with advanced melanoma, 68% of whom had previously received systemic therapy, were observed. The treatment of the patients included increasing doses of nivolumab. Kidney injury occurred in 2 patients, and none of them were classified as grade 3–4 [18]. In the literature, reports of single cases of kidney injury during the use of Nivolumab in the treatment of melanoma can be found. In the described case of a 78-year-old patient, a renal biopsy revealed chronic tubulointerstitial nephritis with acute tubular cell injury. The introduction of steroid therapy resulted in the normalisation of creatinine levels [19]. The best summary of the use of nivolumab monotherapy in melanoma treatment is a study by Weber et al., containing data from two phase I studies and two phase III trials. In the group of 575 patients, only 1.4% experienced renal complications, with grade 3–4 in 0.3% [20].

Another antibody used in cancer therapy is ipilimumab. In the trial conducted by Hodi and co-authors (randomised phase II trial), the effects of ipilimumab alone were compared with Sargramostim: 245 patients were divided into two groups: I (*n* = 123), treated with ipilimumab at a dose of 10 mg/kg body weight with Sargramostim, and II *(n* = 122), treated with ipilimumab at a dose of 10 mg/kg. The median follow-up was 13.3 months. During this period, 1 patient using dual therapy had a grade 3 creatinine elevated level. In the case of monotherapy, 1 patient (1%) showed signs of acute kidney injury in the course of grade 3 nephritis. None of the deaths were caused by kidney injury [21]. The case described by Belliere et al. is the confirmation of the inflammatory aetiology of kidney injury after the use of ipilimumab. A 68-year-old woman, 87 days after the last drug infusion, was admitted to the hospital due to acute kidney injury. A kidney biopsy revealed mild acute tubular injury and inflammatory interstitial fibrosis. Renal function parameters normalised after steroid therapy [22].

One of the therapeutic options in the treatment of melanoma is to combine ipilimumab with nivolumab. Analysing previous studies, the percentage of renal complications during dual therapy can be expected to be higher compared to monotherapy. Sznol et al. published a paper containing data from the phase I, II and III studies to assess the length of survival during the use of dual therapy versus monotherapy. These researchers analysed the safety profile of such treatment: 448 patients were assessed, the median follow-up was 13.2 months, 20 patients (4.5%) experienced renal complications, and 7 (1.6%) of them were classified as grade 3–4 complications. Four out of seven patients received immunomodulating treatment. Patients received at least one dose of nivolumab of 1 mg/kg and ipilimumab of 3 mg/kg every 3 weeks × 4 (induction phase) followed by nivolumab at a dose of 3 mg/kg every 2 weeks (continuation phase). The median time to the onset of symptoms of kidney injury was 16.3 weeks. It was the longest time among all observed complications. In contrast, the time to symptom relief was 1.9 weeks and was the shortest compared to other complications [23]. Murakami and co-authors described the case of a 75-year-old man with metastatic melanoma who developed acute kidney injury and a rash. The patient was treated with nivolumab (1 mg/kg) and ipilimumab (3 mg/kg), and symptoms appeared after the second treatment cycle. A kidney biopsy revealed diffuse interstitial inflammation with moderate interstitial oedema and tubulitis. The patient was initially treated with steroid therapy, but when symptoms recurred, mycophenolate mofetil was added. During the following third hospitalisation, the patient died as a result of bloody diarrhoea. The autopsy showed haemorrhagic colitis [24].

The last discussed medication is pembrolizumab. Ribas et al. published the results of a phase II randomised trial comparing the effects of different doses of pembrolizumab and chemotherapy. Patients had previously been treated with ipilimumab and related adverse reactions had to be reduced to grade 0–1. The trial included 540 patients. Patients were randomly assigned (1:1:1) to a group receiving intravenous pembrolizumab at a dose of 2 mg/kg (180) or 10 mg/kg (181) every 3 weeks, or chemotherapy (179). Renal complications occurred in only 2 patients (with both doses) with grade < 3 [25]. The results of the phase III trial were published by Robert et al., where 834 patients were assigned (1:1:1) to groups of pembrolizumab at a dose of 10 mg/kg every 2 weeks or every 3 weeks, or four cycles of ipilimumab at a dose of 3 mg/kg every 3 weeks. A complication in the form of nephritis of grade < 3 and grade 3–4 occurred in only 1 patient administered pembrolizumab every 3 weeks and in 1 patient administered ipilimumab, respectively [26]. For more information on nephrotoxicity during the use of pembrolizumab, see published case reports. Izzedine and co-authors presented a paper in which they described 4 cases of acute interstitial nephritis with tubulitis and 3 cases of acute tubular injury while using pembrolizumab at a dose of 2 mg/kg [27]. Diffuse active tubulointerstitial nephritis with severe acute tubular cell injury during the use of pembrolizumab was also described by Escandon et al. [19].

### 2.2. Nephrotoxicity in the Immunotherapy of Non-Small Cell Lung Carcinoma

There are two types of lung cancer: small-cell carcinoma (SCLC) and non-small cell carcinoma (NSCLC). NSCLC constitutes approximately 85% of all lung cancer cases. Factors influencing the development of lung cancer are smoking (including passive smoking), air pollution, occupational exposure and genetic determinants [28]. In recent years, the treatment of NSCLC has been revolutionised through the use of tyrosine kinase inhibitors and checkpoint inhibitors. Despite this, lung cancer remains the leading cause of cancer death in the United States [29]. At present, checkpoint inhibitors are used in first-line therapy, both as monotherapy and in combination with chemotherapy, and as a further treatment [30]. The therapy also includes a VEGF inhibitor, also as first-line or second-line therapy, but only in combination with chemotherapy [31].

Gettinger and co-authors published the results of a phase I cohort study of nivolumab in NSCLC treatment: 129 patients who had previously received intensive treatment were enrolled in this study. The medication was administered intravenously at a dose of 1, 3 or 10 mg/kg as a 1 h infusion every 2 weeks in 8-week cycles. Out of all patients, 4 (3.1%) had grade <3 renal complications [32]. The same number of adverse reactions is reported in the study by Rizvi, where 140 patients with stage IIIB or IV NSCLC, with disease progression or recurrence after prior chemotherapy, were enrolled in the study. Among the patients enrolled in the study, 117 people were treated with nivolumab at a dose of 3 mg/kg as an intravenous infusion every 2 weeks. Nephrotoxicity also occurred in 4 patients (3.4%) and in all the patients with grade <3 [33]. Brahmer et al. presented the results of a study in which they compared the effectiveness and safety of nivolumab and doclitaxel therapy. Patients with stage IIIB or IV NSCLC who experienced a relapse after treatment with the platinum-based regimen were enrolled in the study. Among 131 patients administered nivolumab at a dose of 3 mg/kg, 4 patients (3%) experienced adverse reactions related to kidney injury, and 1 patient (1%) developed grade 3–4 tubulointerstitial nephritis. The median time to onset was 24.1 weeks and the median time to symptom relief was 7.9 weeks [34]. Similar results were obtained in a study conducted by Carbone. Out of 271 patients with untreated stage IV or recurrent NSCLC, complications were assessed in 267 people. Patients assigned to the group treated with nivolumab received the medication at a dose of 3 mg/kg. Renal complications occurred in 5 patients (1.9%), including 1 patient (0.4%) with grade 3–4 complications [35]. A study on a smaller group of patients was published by Dumenil and co-authors. They analysed 67 patients from two French hospitals receiving nivolumab at a dose of 3 mg/kg. Nephrotoxicity was observed in 1 patient (1%) with grade <3 [36]. Another study comparing the effectiveness and safety of nivolumab and doclitaxel is the work of Borghaei et al., where 792 patients were enrolled in the study, and 582 were randomised to receive nivolumab at a dose of 3 mg/kg every 2 weeks (*n* = 292) or doclitaxel (*n* = 290): 287 patients were analysed for adverse reactions, renal complications in the form of kidney injury and elevated creatinine levels were reported in 7 patients (2.4%). All complications were classified as grade <3. The median time to symptom onset was 6.7 weeks, while to symptom relief, 10.1 weeks. Out of 287 patients, 1 patient discontinued treatment due to acute kidney injury caused by nivolumab [37]. Single cases of nephrotoxicity caused by nivolumab have also been reported in the literature. In both cases, the biopsy revealed acute tubulointerstitial nephritis. The patients were treated with steroid therapy that led to the improvement of kidney function [38,39].

Pembrolizumab is another antibody used to treat non-small cell carcinoma. In the phase I trial, Garon et al. assessed the effectiveness and safety of the therapy. The expression level of 1 PD-1 ligand was also assessed. The researchers presented data concerning 498 patients, some of whom received pembrolizumab at a dose of 2 mg/kg (*n1* = 6) or 10 mg/kg (*n2* = 287) every 3 weeks, or 10 mg/kg (*n3* = 202) every 2 weeks. Among all patients, complications in the form of acute kidney injury occurred in 1 patient administered a dose of 2 mg/kg (16.7%), and in 1 patient administered a dose of 10 mg/kg every 3 weeks (0.3%). Complications were present in 0.4% of the entire trial group [40]. A phase III trial published by Reck et al. showed that out of 153 patients administered pembrolizumab at a dose of 200 mg, only 1 patient (0.6%) developed grade > 3 nephritis. Previously untreated patients with advanced NSCLC and expressing PD-L1 at the level of at least 50% of neoplastic cells were enrolled in the trial [41]. A study of a larger population was conducted by Mok and co-authors, who compared the application of pembrolizumab monotherapy to carboplatin-based chemotherapy with pemetrexed or paclitaxel. Out of 636 patients, 3 (<1%) developed nephritis, including 1 (<1%) with grade 3–4 [42]. Pembrolizumab can also be used in combination with chemotherapy. Gandhi and co-authors published a study, in which they compared the effects of pembrolizumab in combination with pemetrexed and platinum-based chemotherapy, and the effects of a placebo in combination with pemetrexed and platinum-based chemotherapy. Patients with non-squamous NSCLC without EGFR and ALK mutations were enrolled in the study. The patients were divided in a 2:1 ratio. Adverse reactions were assessed in 405 patients (*n1*) treated with pembrolizumab at a dose of 200 mg and 202 patients (*n2*) treated with a placebo. In the n1 group, 71 patients (2.1%) experienced an increase in creatinine, regardless of its grade. Renal complications occurred in 7 patients (1.7%), including 6 patients (1.5%) with grade >3 [43]. The case of tubulointerstitial nephritis with IgA nephropathy following the use of pembrolizumab was also reported [44].

Bevacizumab is an example of another antibody used in the treatment of NSCLC. Herbst et al. presented interesting results of a phase I/II trial of the combination therapy of bevacizumab with erlotinib. In Phase I, 12 patients received chemotherapy according to the following schedule: *n* = 3 erlotinib 100 mg/day + bevacizumab 7.5 mg/kg, *n* = 3 erlotinib 100 mg/day + bevacizumab 15 mg/kg, *n* = 6 erlotinib 150 mg/day + bevacizumab 15 mg/kg. Seven patients (58%) developed mild proteinuria classified as grade 1–2. In phase II of the study, 40 patients, who were treated with bevacizumab at a dose of 15 mg/kg, including 34 patients with assessed adverse reactions, were observed. It was shown that proteinuria occurred in 3 patients (9%) [45]. Another study was published by Johnson, who compared the effects of carboplatin/paclitaxel (*n1* = 32) with the effects of bevacizumab at a dose of 7.5 mg/kg in combination with carboplatin/paclitaxel (*n2* = 32), or bevacizumab at a dose of 15 mg/kg in combination with carboplatin/paclitaxel (*n3* = 34). Asymptomatic proteinuria occurred in 21 patients (31.8%) treated with bevacizumab, including 7 patients (10.6%) administered at a low dose and 14 patients (21.2%) administered at a high dose. Nephrotic syndrome occurred in 1 patient (1.5%) administered bevacizumab at a dose of 7.5 mg/kg [46]. The study of a larger group of patients was conducted by Sandler et al. Among 427 patients receiving carboplatin, paclitaxel and bevacizumab at a dose of 15 mg/kg, 13 (3%) developed proteinuria: 11 (2.6%) of the cases were classified as grade 3 complication and 2 (0.5%) as grade 4 complication. After the completion of combination therapy, 215 patients continued treatment with bevacizumab as a monotherapy. In 9 patients (4.2%), grade 3 and 4 proteinuria were diagnosed [47]. The assessment of the above treatment regimen was also undertaken by Niho and co-authors, who published data concerning the Japanese population. Among 119 patients treated with carboplatin, paclitaxel and bevacizumab (15 mg/kg), 61 patients (51%) developed proteinuria classified as grade 1/2 complication, and 1 patient (<1%) grade 3 proteinuria [48]. However, in the Chinese population, proteinuria occurred in 6 (4%) of the 140 patients at grade > 3 complications [49].

Patel et al. presented the results of a study evaluating the efficacy and safety of pemetrexed, carboplatin and bevacizumab (at a dose of 15 mg/kg), followed by maintenance treatment with pemetrexed and bevacizumab (at a dose of 15 mg/kg). Patients with stage IIIB, stage IV or relapsed NSCLC cancer who had not previously received treatment were enrolled in the study. Out of 50 patients, 1 (2%) patient developed grade 3 proteinuria [50].

Reck et al. published the results of a phase III study, where three NSCLC treatment regimens were compared. In the first arm, carboplatin with gemcitabine, in the second arm, carboplatin with gemcitabine and bevacizumab at a dose of 7.5 mg/kg, and in the third arm, carboplatin with gemcitabine and bevacizumab at a dose of 15 mg/kg were used. Out of 330 patients treated with low-dose chemotherapy with bevacizumab, 1 patient (<1%) experienced grade ≥ 3 proteinuria, while in high-dose patients (329 patients), 4 people (1%) experienced this complication of ≥3 grade. In total, renal complications were reported in 0.8% of patients administered bevacizumab [51].

### 2.3. Nephrotoxicity in the Immunotherapy of Colorectal Cancer

The incidence of colorectal cancer is steadily increasing worldwide, especially in developing countries. The factors influencing the development of this cancer include obesity, sedentary lifestyle, consumption of red meat, use of alcohol and smoking. In turn, non-modifiable risk factors are hereditary mutations and inflammatory bowel disease [52]. The most common form of therapy is surgical treatment, and the type of surgery performed depends on the location of the lesion and the presence of metastases [53]. In recent years, immunotherapy has played an increasingly important role. Currently, it can be used both as a first-line therapy in metastatic colorectal cancer and in chemotherapy-resistant tumours characterised by microsatellite instability (MSI-H) and/or abnormalities in DNA repair genes (dMMR) [54].

Overman and co-authors published a paper concerning the treatment of colorectal cancer with nivolumab at a dose of 3 mg/kg every 2 weeks: 74 people were enrolled, and most of them had previously been treated with ≥3 therapies. In total, renal complications occurred in 3 (4%) patients: 2 (2.7%) of them experienced a reaction classified as grade 3–4 due to elevated creatinine levels and acute kidney injury, and 1 patient (1.4%) discontinued the study because of acute kidney injury [55]. The same researcher presented the results of a phase II study of the treatment of colorectal cancer with nivolumab and ipilimumab in patients with dMMR/MSI-H. Patients were administered nivolumab at a dose of 3 mg/kg and ipilimumab at a dose of 1 mg/kg every 3 weeks for four doses, followed by nivolumab at a dose of 3 mg/kg every 2 weeks. Out of 119 patients, 6 (5%) developed renal complications, including 2 (2%) who developed acute kidney damage classified as grade 3-4 complications. Median time to onset of symptoms was 12.6 weeks for all grades. It was the longest period among all observed complications. Patients with acute kidney injury received steroid therapy in accordance with the protocol [56].

Tabernero and co-authors published the results of a phase III RAISE study. It compared the effects of ramucirumab combined with the FOLFIRI regimen (leucovorin, fluorouracil and irinotecan) and FOLFIRI alone. A total of 1072 patients with metastatic colorectal cancer and disease progression during or after first-line treatment (bevacizumab, oxaliplatin and fluoropyrimidine) were enrolled in the study. Patients received either ramucirumab at a dose of 8 mg/kg or a placebo every 2 weeks followed by the FOLFIRI regimen. Adverse events were assessed in 529 patients receiving immunotherapy. Proteinuria developed in 90 patients (17%): grade 1–2 in 74 patients (14%), grade 3 in 15 patients (3%) and grade 4 in 1 patient (<1%). Moreover, 18 (3.4%) cases of acute kidney injury: 11 (2%) of grade 1–2, 6 (1%) of grade 3 and 1 (<1%) of grade 4, were reported [57]. Obermannová et al. interpreted the results of the RAISE study by analysing individual age groups. The researchers divided the study population into people of ≥65 years of age (*n* = 209), <65 years of age (*n* = 320), ≥75 years of age (*n* = 51) and <75 years of age (*n* = 479). Nephrotoxicity in the form of proteinuria of grade ≥ 3 was most common in people < 65 years of age, which constituted 11 patients (3.4%). Grade ≥3 acute kidney injury was most common in the age group ≥75 years, which constituted 3 patients (5.9%) [58].

Another antibody that is used in combination with standard chemotherapy is bevacizumab. Kabbinavar et al. designed a study in which they compared the effects of fluorouracil- and leucovorin-based chemotherapy with a combination therapy with bevacizumab (5 mg/kg), fluorouracil and leucovorin. Patients considered as non-optimal candidates for irinotecan in first-line treatment were enrolled in the experiment. Adverse reactions in the bevacizumab group were assessed in 100 patients. Proteinuria occurred in 38 patients (38%), including grade 2 in 7 patients (7%) and grade 3 in 1 patient (1%) [59]. Emmanouilides et al. described the effectiveness of treatment with bevacizumab (5 mg/kg), oxaliplatin, leucovorin and fluorouracil (FOLFOX-4): 53 patients were enrolled in the study. Proteinuria was observed in 11 patients: grade 1 in 7 patients (13%) and grade 2 in 4 patients (7.5%) [60]. Interesting conclusions can be drawn from the work of Hurwitz and co-authors. The researchers compared the effects and effectiveness of irinotecan-, fluorouracil- and leucovorin-based chemotherapy (FOLFIRI) to FOLFIRI with bevacizumab (5 mg/kg). Among 393 patients assessed for adverse reactions, proteinuria occurred in 104 patients (26.5%), including grade 2 in 12 patients (3.1%) and grade 3 in 3 patients (0.8%). Interestingly, these values are similar to those observed in the group of 397 people treated with a placebo. In that group, proteinuria occurred in 21.7% of patients, including grade 2 in 5.8% and grade 3 in 0.8% [61]. Saltz presented the results of a phase III study. He described the effectiveness of the XELOX (capecitabine, oxaliplatin) and FOLFOX-4 (fluorouracil, folinic acid, oxaliplatin) chemotherapy in combination with bevacizumab (the dose applied with XELOX was 7.5 mg/kg, while with FOLFOX-4, 5 mg/kg) or without it. Out of 694 patients administered the bevacizumab regimen, grade 3/4 proteinuria occurred in 4 patients (<1%) [62].

## 3. Nephrotoxicity in Chemotherapy 

The use of chemotherapy in the treatment of neoplasms started at the beginning of the 20th century. Initially, this field was dominated by surgery and radiation therapy. It was only after the importance of micro-metastases and their role in the treatment process was noticed, that the idea of using adjuvant chemotherapy was born. From that moment on, combination therapy based on combining these three methods in a way that maximises the anti-cancer effect and minimises the toxic effect on healthy tissues has become the basis of clinical management [63]. Chemotherapeutic agents can affect any element of the nephron, leading to the appearance of clinical symptoms such as proteinuria, hypertension, electrolyte disorders, glomerulonephritis, acute and chronic interstitial nephritis and acute kidney injury. Numerous medication-related renal complications do not have a well-defined pathomechanism, which makes it difficult to develop strategies to prevent or minimise their incidence [6]. The IRMA study showed that kidney injury is common in people taking anti-cancer therapy and leads to the necessity of dose adjustment in these people [64]. In this review of the literature, the authors will try to present some of the renal complications caused by chemotherapy.

### 3.1. Nephrotoxicity in the Chemotherapeutic Treatment of Colorectal Cancer

Chemotherapy in the treatment of colorectal cancer is an adjuvant therapy. It is used mainly in the case of the third stage of development. The decision to include it in stage II treatment should be made individually, based on the assessment of the risks and benefits of the decisions [65]. In this paper, selected chemotherapy regimens for the treatment of colorectal cancer will be presented.

José Luis Fírvida and co-authors presented a study, in which the safety and effectiveness of irinotecan (CPT-11) in the first-line therapy of patients with advanced colorectal cancer were assessed. The analysis included patients who had not previously been treated with chemotherapy or who had received adjuvant chemotherapy. The dose of 350 mg/m^2^ of CPT-11 was used every 3 weeks. Out of the 65 patients, 6 (9.2%) developed renal insufficiency. During the study, 1 patient died as a result of acute kidney injury accompanied by neutropenia [66]. Rödel et al. in their study assessed the safety of preoperative radiation therapy (RT) with the simultaneous use of capecitabine and oxaliplatin (XELOX-RT) and the inclusion of four cycles of adjuvant XELOX chemotherapy in patients with rectal cancer. The following regimen was used: preoperative radiation therapy (50.4 Gy in 28 fractions), capecitabine at a dose of 1650 mg/m^2^, oxaliplatin at a dose of 50 mg/m^2^. The surgery was scheduled for 4 to 6 weeks after the end of XELOX-RT. This was followed by four cycles, including capecitabine at a dose of 1000 mg/m^2^ twice daily, and oxaliplatin at a dose of 130 mg/m^2^. Among 104 patients who received neoadjuvant chemotherapy, 17 patients (16%) experienced an increase in creatinine levels, including 16 cases (15%) which were classified as grade 1/2 and 1 case (1%) classified as grade 3. Grade 1/2 proteinuria occurred in 12 patients (12%). Complications after adjuvant chemotherapy were assessed in 73 patients: 13 patients (18%) experienced elevated creatinine levels, while 12 patients (16%) had grade 1/2 proteinuria [67]. Another phase II study evaluating the toxicity of chemotherapy is a study published by Willeke et al. The researchers evaluated neoadjuvant therapy for rectal cancer based on irinotecan (50 mg/m^2^) in combination with capecitabine (500 mg/m^2^) with concomitant radiotherapy (CapIri-RT). Nephrotoxicity in the form of an increase in creatinine levels was reported in 3 (8.3%) out of 36 patients [68]. Wilke and co-authors presented the results of the large international MABEL study. The treatment included patients with metastatic colorectal cancer expressing epidermal growth factor, in whom the treatment with a regimen containing irinotecan was not successful. In the study, cetuximab (the initial dose of 400 mg/m^2^ once a week, then 250 mg/m^2^) in combination with various doses of irinotecan (125 mg/m^2^ weekly for 4 weeks followed by 2 weeks with no medication, or 180 mg/m^2^ every 2 weeks or 350 mg/m^2^ every 3 weeks) was used. One death associated with the use of irinotecan in a regimen of 350 mg/m^2^ every 3 weeks, caused by acute kidney injury, was reported [69].

Cassidy et al. published interesting results of a study concerning the use of capecitabine (1250 mg/m^2^ twice a day) or 5-fluorouracil (425 mg/m^2^) with leucovorin (20 mg/m^2^). In the observed group of patients, 596 vs. 593, no renal complications were found. The researchers focused on assessing the occurrence of adverse reactions depending on baseline creatinine clearance (ml/min). In the observed group of patients, approximately 45% of patients had normal renal function (creatinine clearance > 80 mL/min), another 45% had mild renal insufficiency (51–80 mL/min) and 10% had moderate disorders (30–50 mL/min). It was observed that in both arms of the study, the incidence of grade 3 or 4 adverse reactions was higher in patients with moderate renal insufficiency than in patients with normal renal function. In the case of the capecitabine regimen, it concerned 54%, and in the case of the 5-fluorouracil regimen, 51% of patients. Similar relationships were found in the case of the necessity to reduce the dose, the highest percentage of patients concerned those with a clearance in the range of 30–50 mL/min, 44% and 52%, respectively [70].

Information on the nephrotoxicity of oxaliplatin can also be found in the literature. In the first case report, a 52-year-old patient developed symptoms of haemolytic-uraemic syndrome with anuria and acute kidney injury. After the applied treatment based on intensive hydration, administration of high doses of corticosteroids and diuretics, renal and haematological parameters normalised [71]. Another report concerns a 65-year-old man with advanced colorectal cancer, who developed anuria, acute kidney injury and thrombocytopenia after 5 cycles of chemotherapy. The patient required haemodialysis. The performed biopsy revealed features of acute tubular necrosis [72].

### 3.2. Nephrotoxicity in the Chemotherapeutic Treatment of Non-Small Cell Lung Carcinoma

Most of the newly diagnosed lung cancers are non-small cell lung carcinomas (NSCLCs). More than half of them at diagnosis are locally advanced (stage III). Chemoradiation therapy remains the standard of care in the management of stage IIIB or IIIA patients with unresectable or inoperable disease [73]. Platinum-based chemotherapy remains an important part of the treatment of locally advanced NSCLC because, according to the conducted studies, it improves survival in patients with tumours considered both resectable and unresectable [74]. Stage III NSCLC is currently considered a very heterogeneous disease, which requires multidisciplinary management [75].

Asahina et al. presented the results of a phase II study assessing the effectiveness and safety of gefitinib (250 mg) as first-line therapy in advanced non-small cell lung carcinoma with EGFR mutations. The observations were made on the Japanese population. Out of 16 patients, only 2 (13%) developed grade 1 elevated creatinine levels [76]. Similar results were obtained in a study by Tamura et al.; in this multicentre study, 5 patients (17.8%) had elevated creatinine levels [77]. Other observations of renal complications were presented by D’Addario and co-authors. Among 61 patients, renal insufficiency classified as grade 3 was reported in 1 patient (1.64%) [78]. In a phase III study, Maemondo et al. compared the safety profile of gefitinib (250 mg) to standard carboplatin- and paclitaxel-based chemotherapy (200 mg/m^2^). In patients treated with gefitinib (*n* = 114), a grade I creatinine level increase was observed in 3 patients (2.63%). In the comparative population (*n* = 113), creatinine levels increased in 13 patients (11.5%), including 12 cases (10.6%) of stage I and 1 case (0.88%) of stage 2 [79]. 

Gatzemeier et al. presented the results of a study comparing the safety and effectiveness of the therapy with gemcitabine (1250 mg/m^2^) and cisplatin (80 mg/m^2^) in combination with erlotinib (150 mg/day) or a placebo. Renal insufficiency was more common in the erlotinib group (5%) compared to the placebo group (1%). Moreover, 2 deaths caused by renal insufficiency possibly related to the use of erlotinib were recorded [80]. In a study conducted by Jänne and co-authors, no increased incidence of renal complications was observed in either the group administered erlotinib (150 mg/day) or the group administered erlotinib (150 mg) in combination with paclitaxel (200 mg/m^2^) and carboplatin. In the triple treatment group, 1 death (1%) caused by renal insufficiency took place [81]. A group of French researchers published the results of a phase III study, in which they compared the effectiveness of continued treatment with gemcitabine (1250 mg/m^2^) or erlotinib (150 mg/day) after an induction cycle consisting of cisplatin (80 mg/m^2^) and gemcitabine (1250 mg/m^2^). The control group consisted of people who were followed up after the initial treatment. In the case of progression, patients in all 3 arms of the study received treatment with pemetrexed (500 mg/m^2^). The highest number of renal complications was reported in the group receiving erlotinib-in 8 patients (5.2%), including 2 (1.3%) who were classified as grade 3/4. In the case of patients using gemcitabine, 7 cases (4.5%) of renal insufficiency were reported, including 1 case (0.6%) of grade 3/4. Renal complications were also recorded in the control arm (in 2 patients, 1.3%) [82].

Rosell et al. proved that therapy with paclitaxel (200 mg/m^2^) and cisplatin (80 mg/m^2^) causes more complications related to nephrotoxicity than therapy with paclitaxel (200 mg/m^2^) and carboplatin (12.6% vs. 4.9%) [83]. Similar conclusions can be drawn from the study of Schiller and co-authors. In patients administered paclitaxel (135 mg/m^2^) and cisplatin (75 mg/m^2^), 1% of grade 3 renal complications was recorded, and in the case of carboplatin and paclitaxel (225 mg/m^2^), this proportion was 0.34%. In this study, the largest number of adverse reactions were reported in the cisplatin (100 mg/m^2^) and gemcitabine (1000 mg/m^2^) groups, in 9 patients (3%), including 3 patients (1%) classified as grade 4/5 [84].

In the case of the application of doclitaxel (25 mg/m^2^) with cisplatin (20 mg/m^2^) in people ≥ 75 years of age in the Japanese population, renal complications were shown to be rare and mild (in 1 patient out of 33 patients, grade 2) [85]. Similarly, during the use of higher doses of doclitaxel (75 mg/m^2^) and cisplatin (75 mg/m^2^) in various age groups, nephrotoxicity was relatively rare (1%), however, complications were classified as grade III in all 3 patients [84]. Another regimen that can be used in non-small cell lung carcinoma is the combination of vinorelbine (25 mg/m^2^) with cisplatin (50 mg/m^2^). The analysed studies showed that elevated creatinine levels occurred in 37 patients (16%) [86], while no renal complications were recorded in the work of Kreuter et al. [87]. Treatment of extensive neoplasms may be based on the use of topotecan or etoposide in combination with cisplatin. The study that best summarises the treatment options for lung cancer is the analysis carried out by Japanese researchers. Ohe et al. compared the effectiveness and toxicity of the following regimens: cisplatin (80 mg/m^2^) with irinotecan (60 mg/m^2^), carboplatin with paclitaxel (200 mg/m^2^), cisplatin (80 mg/m^2^) with gemcitabine (1000 mg/m^2^) and cisplatin (80 mg/m^2^) with vinorelbine (25 mg/m^2^). Among the described treatment algorithms, the largest number of renal complications in the form of an increase in creatinine levels were observed in the group using irinotecan with cisplatin (*n* = 147), it was 9 patients (6.1%). The second algorithm in terms of the frequency of renal complications is the combination of cisplatin with vinorelbine (*n* = 146), 9 patients (6.2%), and then cisplatin with gemcitabine (*n* = 151), 7 patients (4.6%). The fewest complications were reported in the group of patients administered carboplatin with paclitaxel (*n* = 148), 2 patients (1.4%); however, 1 death (0.7%) as a result of renal insufficiency was recorded in this population [88].

## 4. Management and Treatment of Nephrotoxicity during Immunotherapy and Chemotherapy

Immune-related nephritis or other renal dysfunction may occur during nivolumab, pembrolizumab and ipilimumab treatment. The algorithm is the same for each of these drugs. Before each administration of the above-mentioned substances, sodium, potassium, creatinine and urea levels should be determined. In a situation of an increase in creatinine as described in grade 1, treatment should be continued according to the protocol and creatinine levels should be measured weekly. Additionally, the patient’s hydration status should be assessed, potentially nephrotoxic drugs should be discontinued, the urine protein to creatinine ratio should be determined, and if a urinary tract infection is suspected, urinalysis and urine culture should be ordered. Kidney ultrasound or ultrasonographic assessment of renal arteries (Doppler ultrasound) can be done to rule out changes in the renal vessels. If the creatinine concentration returns to normal, follow-up measurements are made according to the protocol. In grade 2, it is necessary to delay the use of the drug and monitor creatinine levels every 48–72 h. If an immune-related adverse event is suspected, administration of 0.5 to 1.0 mg/kg/day of methylprednisolone intravenously or the equivalent of this dose orally is required. Also, a renal biopsy should be considered. Additional tests, such as in grade 1, should be done, as well as measurements of ANA (antinuclear antibody), complement C3, C4, ANCA (anti-neutrophil cytoplasmic antibody), anti-GBM (anti-glomerular basement membrane), hepatitis B and C, HIV, immunoglobulins and protein electrophoresis, 24 h urine collection (evaluation of proteinuria). When complications classified as grade 3 occur, the administration of drugs should be delayed, the concentration of creatinine should be measured daily, the patient should be consulted with a nephrologist, treatment with methylprednisolone at a dose of 1.0–2.0 mg/kg should be started and additional tests, as in grade 1 and grade 2, should be performed. Steroid therapy is recommended until creatinine values are obtained as in grade 1. Weaning of methylprednisolone should last 2 to 4 weeks for grade 2 and ≥4 weeks for grade 3–4 [89].

Proteinuria monitoring with urine dipstick testing is necessary during treatment with bevacizumab or ramucirumab. Patients with a score of 2+ or higher should undergo further evaluation with a 24 h urine collection (evaluation of proteinuria). Medications should be withheld if proteinuria is ≥2.0 g/24 h, while continuation of this treatment is possible if proteinuria is <2.0 g/24 h. Re-inclusion of bevacizumab is associated with the use of a reduced dose (with the initial dose of 8 mg/kg reduction to 6 mg/kg, with the initial dose 10 mg/kg reduction to 8 mg/kg). Another episode of proteinuria > 2.0 g/24 h and then its normalisation requires another dose reduction (from 6 to 5 mg/kg, from 8 to 6 mg/kg). Permanent discontinuation of bevacizumab treatment occurs when nephrotic syndrome develops (proteinuria > 3.5 g/24 h with associated oedema and arterial hypertension). Permanent discontinuation of ramucirumab occurs if there is a proteinuria > 3.0 g/24 h or if nephrotic syndrome occurs [90].

Chemotherapy can lead to acute kidney injury. Proper hydration is a commonly used and recommended method to prevent nephrotoxicity. Increase in serum creatinine during chemotherapy may lead to a reduction of the dose or even to temporary or permanent discontinuation of the drug. Table 1 presents recommendations for dosage adjustments of selected chemotherapeutic agents in relation to creatinine clearance based on medicinal products characteristics on the Food and Drug Administration (FDA) website and on the existing treatment protocols.

## 5. Conclusions

Over the recent years, progress has been made in the diagnosis and treatment of cancer. It has significant effects on prolongation of survival in oncological patients. The treatment used in oncology—chemotherapy and immunotherapy—may, however, result in complications, including nephrotoxicity. Renal complications may force reduction of the dose of the medication, modification of the therapy or permanent disqualification from a given treatment regimen. Predicting possible side effects is much more difficult with chemotherapeutic agents than with immunotherapeutic agents due to limited data on the pathomechanisms of kidney damage in this mechanism. 

Analysing the data from the literature, when two immunotherapeutic agents are used together, the number of recorded renal complications increases (nivolumab vs. ipilimumab vs. nivolumab + ipilimumab in melanoma 1. 57% vs. 0. 59% vs. 4. 46%). Moreover, the cited case reports may suggest a more severe course of these complications in patients taking these drugs together. The dose of the medicine used and the treatment regimen also play an important role. Investigating the adverse events occurring in patients treated with bevacizumab, it is clear that the most cases of nephrotoxicity were recorded at the dose of 15 mg/kg compared to 7.5 mg/kg in non-small cell lung cancer. Moreover, bevacizumab and ramucirumab are the cause of the largest number of renal complications among immunotherapeutic agents described above. However, it should be noted that these drugs are not used in monotherapy, but only in combination with chemotherapy, which also has a negative effect on the kidneys. Cisplatin is the best-described substance with the greatest nephrotoxic potential among the chemotherapeutic agents. This drug is used in many regimens of cancer treatment and it shows high effectiveness. Despite numerous studies on the mechanism and prevention of AKI during such treatment, it is not possible to obtain an unambiguous strategy for the prevention of AKI that can be used in oncological treatment at this moment [91]. In this article, we presented numerous treatment regimens in which cisplatin in different doses and in combination with other chemotherapeutic drugs causes acute kidney injury. Simultaneously, it is a substance that quite quickly requires dose reduction if the creatinine clearance is reduced. Cancer stage and previous chemotherapy are also crucial for renal complications. However, it should be mentioned that in large groups of patients, the progression of the disease per se leads to cachexia, dehydration [92] and kidney damage. Moreover, there are often other risk factors of AKI such as age, comorbidities and medications used.

Therefore, it is important to carry out studies on the effects of different substances, such as chemotherapeutics and immunotherapeutics, on renal function. It is also necessary to develop common standards to facilitate cooperation between oncologists and nephrologists both during and after treatment of the patient with cancer. The ability to estimate the risk of particular renal complications and its possible prevention may affect the effectiveness of the therapeutic process. Therefore, it is necessary to conduct further research and develop algorithms for management of a cancer patient, especially during such intensive progress in oncology.

## Figures and Tables

**Table 1:** Drug dosage adjustments based on creatinine clearance.

Chemotherapeutic Agent	Dosage
Capecitabine	≥50 mL/min—no dosage modification 30–49 mL/min—75% of the dose should be used <30 mL/min—the drug should be discontinued
Carboplatin	41–59 mL/min—an initial dose of 250 mg/m^2^ should be used 16–40 mL/min—an initial dose of 200 mg/m^2^ should be used
Cisplatin	>60 mL/min—no dose reduction is required (in most protocols) 45–59 mL/min—75% of the dose should be used <45 mL/min—the inclusion of carboplatin should be considered
Gefitinib	>20 mL/min—no dose reduction is required
Gemcitabine	<30 mL/min—reduction of the dose or discontinuation of the drug is required
Oxaliplatine	>30 mL/min—no dose reduction is required <30 mL/min—the drug should be discontinued
Paclitaxel	30–90 mL/min—no dosage modification is required
Pemetrexed	<45 mL/min—a dose reduction should be considered
